# Association between higher estimated glucose disposal rate and reduced prevalence of hyperuricemia and gout

**DOI:** 10.3389/fnut.2025.1658286

**Published:** 2025-09-23

**Authors:** Pengfei Liu, Kaisaierjiang Kadier, Xiaoli Wu, Xiangyu Sun, Ge Zhang, Xiaozhu Liu, Daqing Song, Chunying Cui

**Affiliations:** ^1^Department of Cardiology, Affiliated Hospital of Jining Medical University, Jining, Shandong, China; ^2^Shandong Provincial Key Medical and Health Discipline of Cardiology (Affiliated Hospital of Jining Medical University), Shandong, China; ^3^Department of Cardiology, First Affiliated Hospital of Xinjiang Medical University, Urumqi, China; ^4^Clinical Medicine College, Xinjiang Medical University, Urumqi, China; ^5^Department of Cardiology, First Affiliated Hospital of Zhengzhou University, Zhengzhou, China; ^6^Department of Critical Care Medicine, Beijing Shijitan Hospital, Capital Medical University, Beijing, China; ^7^Emergency Department, Jining No. 1 People’s Hospital, Jining, China

**Keywords:** estimated glucose disposal rate, hyperuricemia, gout, metabolism disorders, diabetes, NHANES

## Abstract

**Objective:**

Insulin resistance (IR) is closely associated with hyperuricemia (HUA) and gout; however, the relationship between the estimated glucose disposal rate (eGDR), a novel comprehensive indicator of systemic IR, and the prevalence of HUA and gout in the general population remains unclear.

**Methods:**

This study analyzed data from 29,340 participants included in the National Health and Nutrition Examination Survey (NHANES) database. Multivariable logistic regression models and restricted cubic splines were employed to assess the association between eGDR and HUA and gout. Subgroup analyses were conducted to examine potential variations in the findings across different subgroups stratified by age, sex, race, and diabetes status.

**Results:**

The prevalence rates of HUA and gout among participants were 17.51 and 3.95%, respectively. Fully adjusted multivariable logistic regression models revealed that for each 1-unit increase in eGDR, the prevalence of HUA decreased by 17% (OR: 0.83, 95% CI: 0.81–0.85, *p* < 0.001), and the prevalence of gout also decreased by 17% (OR: 0.83, 95% CI: 0.79–0.87, *p* < 0.001). Compared to participants with eGDR < 4 mg/kg/min, those with eGDR levels of 4–6, 6–8, and > 8 mg/kg/min exhibited significantly lower OR values for both HUA and gout. Furthermore, subgroup analyses for HUA demonstrated significant interaction effects between eGDR and age, sex, race, and diabetes status (*p* < 0.05), while subgroup analyses for gout indicated significant interactions between eGDR and age and diabetes status (*p* < 0.05).

**Conclusion:**

A significant inverse association was observed between eGDR and the prevalence of HUA and gout. Maintaining higher levels of eGDR plays a positive role in reducing the risk of HUA and gout in the general population. Moreover, this association was particularly pronounced in middle-aged and younger populations as well as in non-diabetic individuals.

## 1 Introduction

Hyperuricemia (HUA) is a metabolic disorder characterized by persistently elevated serum uric acid (SUA) concentrations exceeding the physiological threshold. Its pathogenesis is primarily associated with purine metabolism disorders or impaired SUA excretion. Epidemiological surveys indicate that the global prevalence of HUA has significantly increased in recent years, with approximately 20.1% of adults in the United States affected by HUA (1). Furthermore, the prevalence of HUA in Asian populations has reached 13.3 to 20.1%, making it the second most common metabolic disorder after diabetes (2–4). Studies show that about 10–20% of patients with HUA get gout (5). Gout is an inflammatory joint disease caused by HUA. It happens when monosodium urate crystals build up in joints and soft tissues (6). The worldwide prevalence of gout in adults varies between 0.68 and 3.90% (7). Recently, the rate of gout has been gradually rising. This increase could be associated with shifts in the age distribution of the population. It may also relate to a higher number of cases of metabolic syndrome and other related metabolic disorders (8). Glucose metabolism is strongly connected to SUA metabolism. One important feature of disorders in glucose metabolism is IR (9). Studies suggest that higher oxidative stress and systemic chronic low-grade inflammation in HUA contribute to reduced insulin sensitivity. This reduction results in increased IR (10). At the same time, elevated IR causes higher insulin levels. Increased insulin promotes the kidneys to reabsorb more uric acid. This process further raises serum uric acid levels (11, 12). Lowering IR can effectively decrease SUA levels and reduce the risk of gout (13, 14). Clinical data indicate that individuals with HUA have a 10 to 12% greater risk of developing type 2 diabetes compared to the general population. Furthermore, the prevalence of HUA among patients with diabetes ranges from 20.5 to 28.1% (15).

The hyperinsulinemic-euglycemic clamp technique is the gold standard for diagnosing IR. However, this method is complex and invasive, limiting its application in clinical practice (16). The estimated glucose disposal rate (eGDR) is a comprehensive marker of insulin sensitivity and long-term blood glucose regulation. It incorporates standard clinical variables, including waist circumference, hypertension status, and hemoglobin A1c (HbA1c) levels. Studies have shown that eGDR is negatively correlated with IR levels and is considered a reliable alternative indicator of IR (17). The relationship between eGDR and both HUA and gout remains poorly defined. This study aims to examine the association between eGDR and the prevalence of HUA and gout. Through this, it seeks to clarify the potential link between IR and these disorders. Additionally, patients will be stratified based on their eGDR levels. This approach is intended to establish a scientific foundation for the risk stratification and clinical management of HUA and gout in clinical practice.

## 2 Materials and methods

### 2.1 Study design and population

Data from six cycles (2007–2018) of the National Health and Nutrition Examination Survey (NHANES) were analyzed secondarily in this study. NHANES provides nationally representative U.S. population surveillance through integrated data collection capturing sociodemographic characteristics, clinical histories, validated dietary measures, and clinically assessed biomarkers. This survey has been extensively used in public health research. The survey and research protocols of NHANES were approved by the Ethics Review Board of the National Center for Health Statistics (NCHS). This approval ensured that the study adhered to ethical standards. Written informed consent was obtained from all participants. Further NHANES methodological details are accessible via https://www.cdc.gov/nchs/nhanes/index.htm.

**Figure 1:**
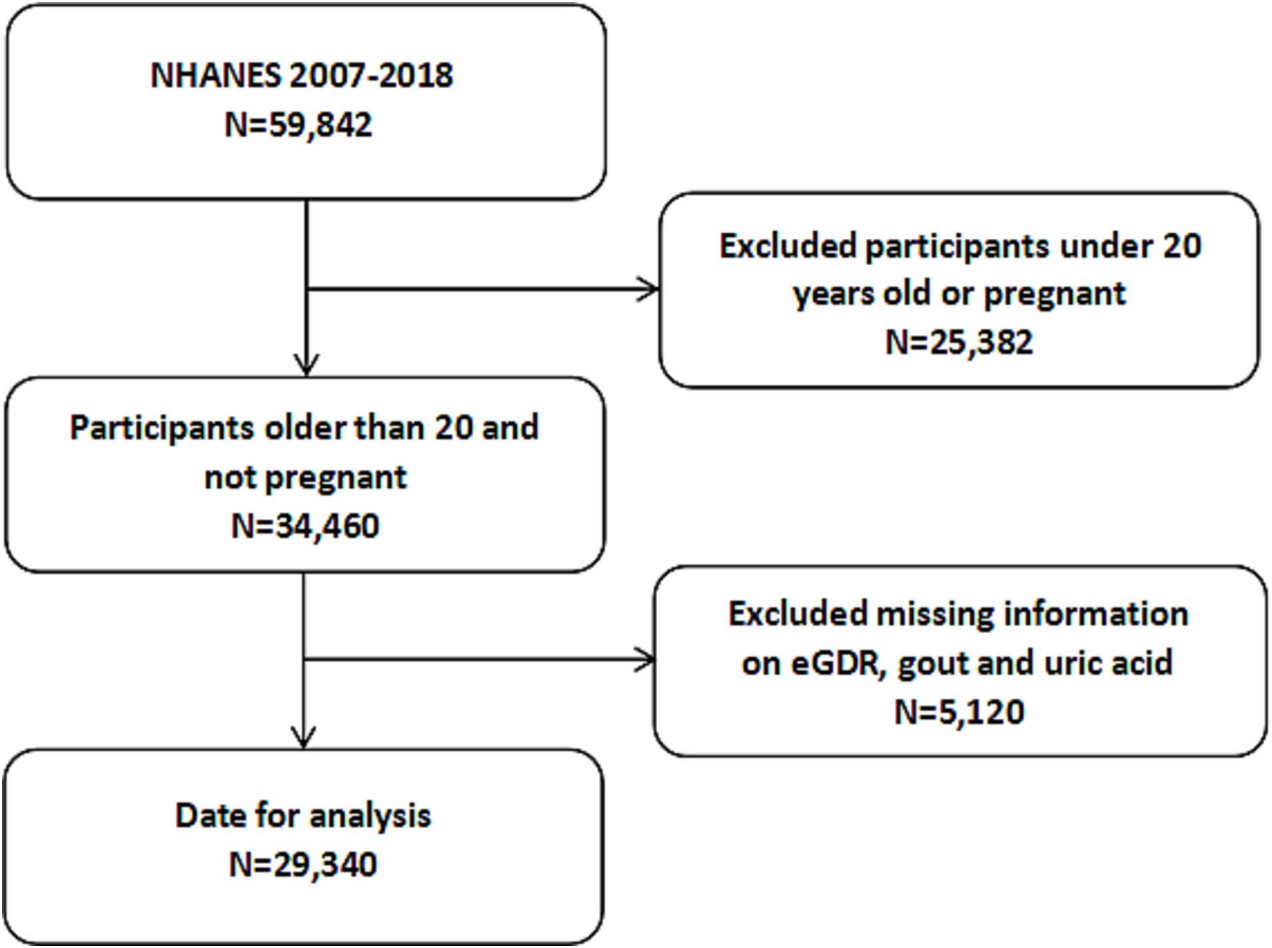
Flowchart of the participants selection from NHANES 2007–2018. NHANES, National Health and Nutrition Examination Survey.

From 2007 to 2018, a total of 59,842 participants were enrolled in the NHANES survey. Initially, we excluded individuals younger than 20 years of age and those who were pregnant (*N* = 25,382). Additionally, we further excluded 5,120 participants due to missing data on eGDR, gout, or uric acid levels. Consequently, a total of 29,340 participants was included in the analysis. The detailed screening process is illustrated in Figure 1.

### 2.2 Calculation of eGDR

The exposure variable in this study was the estimated glucose disposal rate (eGDR). The formula for calculating eGDR was as follows (18): eGDR (mg/kg/min) = 21.158 − (0.09 × waist circumference WC) − (3.407 × hypertension) − (0.551 × HbA1c), where WC represents waist circumference (cm), hypertension is coded as yes = 1 or no = 0, and HbA1c is expressed as a percentage (%). Information on hypertension was obtained through self-reported physician diagnosis, current use of antihypertensive medication, or an average blood pressure measurement of ≥140/90 mmHg.

### 2.3 Diagnosis of HUA and gout

Serum samples were transported to the National Center for Environmental Health and stored at −30 °C until they were delivered to the laboratory for the measurement of serum uric acid levels. HUA was defined based on sex-specific criteria: >7.0 mg/dL for males and >6.0 mg/dL for females (19). During the home interviews, all participants were asked the question “Has a doctor or other healthcare professional ever told you that you have gout?” Those who answered “yes” were defined as having gout (19). Self-reported disease diagnoses have been widely utilized in epidemiological research (20).

### 2.4 Covariates

The NHANES database provides comprehensive demographic and health-related data (14), including age (20–39, 40–59, and ≥60 years), sex (male and female), race (Non-Hispanic White, Mexican American, Non-Hispanic Black, and others), education (less than high school, high school, and above high school), marital status (married/living with partner, widowed/divorced/separated, and never married), and poverty-income ratio (PIR; <1.3, 1.3–3.5, and >3.5). Participant health statuses encompass smoking status (never, now and former), alcohol consumption (never, former, mild–moderate and heavy), physical activity levels (inactive, low-active, highly active, and extremely highly active), body mass index (BMI), high-density lipoprotein cholesterol (HDL-C), triglyceride levels, random blood glucose, and the presence or absence of chronic kidney disease (CKD), diabetes mellitus (DM), cancer, and cardiovascular disease (CVD). For detailed definitions, please refer to Supplementary Table 1.

### 2.5 Statistical analyses

All statistical analyses in this study were conducted in accordance with the Strengthening the Reporting of Observational Studies in Epidemiology (STROBE) Statement: guidelines for reporting observational studies (21). Given the complex, multistage, stratified probability sampling design employed by the NHANES, this study incorporated survey design variables and sampling weights to minimize analytical bias and ensure accurate estimates. Continuous variables were expressed as means with standard deviations (SD), while categorical variables were described using weighted percentages with 95% confidence intervals (95% CI). Differences between groups were assessed using one-way analysis of variance for continuous variables and Rao-Scott chi-square tests for categorical variables.

The association between eGDR and HUA and gout was assessed in this study using weighted logistic regression models, with eGDR incorporated both as a continuous variable and as a categorical variable. Model 1 the crude model. Model 2 further adjusted for age, sex and race. Model 3 further adjusts for education, marital status, PIR, smoking status, alcohol consumption, physical activity levels, BMI, HDL-C, triglyceride levels, random blood glucose, DM, CKD, cancer and CVD on the basis of Model 2. Furthermore, restricted cubic spline (RCS) regression with 3 knots positioned at the 10th, 50th, and 90th percentiles was employed to examine the potential nonlinear relationship between eGDR and HUA and gout.

To explore the relationship between eGDR and HUA and gout across diverse populations, subgroup analyses were conducted based on age, sex, race, and DM status within fully adjusted models. Multiplicative interactions were evaluated using the likelihood ratio test. Missing covariate data were addressed through imputation via the MissForest software package (22). Sensitivity analyses were performed under three conditions: (1) restricting the analysis to participants with complete covariate data, (2) excluding individuals with CVD and cancer, and (3) employing uncomplicated sampling procedures. All statistical analyses were carried out using R version 4.2.2 (R Foundation for Statistical Computing, Vienna, Austria). Two-sided statistical tests were applied, with a significance threshold of *p* < 0.05.

## 3 Results

### 3.1 Baseline characteristics

A total of 29,340 NHANES participants (mean age 47.48 ± 0.23 years, 50.90% female [95% CI:48.58-53.22%]) were analyzed. The cohort showed a mean serum uric acid level of 5.43 ± 0.01 mg/dL. Prevalence rates included 3.95% (95% CI: 3.58-4.33%) of gout and 17.51% (95% CI:16.46-18.55%) HUA among this population. Moreover, the DM prevalence was 12.43% (95% CI:11.74-13.12%). Participants who showed high eGDR were more likely to be younger and female, compared to those with low eGDR levels. These participants were characterized by a higher level of education and household income. They were also less likely to smoke and drink as often, were more physically active, and had a higher HDL-C. Furthermore, they had generally low levels of serum uric acid and HbA1c levels. The high eGDR cohort also had significant reduction in prevalence of CKD, gout, HUA, CVD, hypertension, and DM. Particularly, participants with eGDR >8 mg/kg/min had the most favorable disease profiles - demonstrating significant lower prevalence of HUA: 9.57% (95% CI:8.81-10.33%) and gout: 1.36% (95% CI:1.10-1.61%). All of the baseline characteristics have been outlined in Table 1.

**Table 1:** Basic characteristics of study participants from NHANES 2007–2018.

Characters	Overall (*N* = 29,340)	eGDR < 4 (mg/kg/min) (*N* = 3,241)	eGDR 4–6 (mg/kg/min) (*N* = 6,257)	eGDR 6–8 (mg/kg/min) (*N* = 5,347)	eGDR > 8 (mg/kg/min) (*N* = 14,496)	*p*-value
Age-year	47.48 ± 0.23	55.98 ± 0.31	56.82 ± 0.30	52.02 ± 0.34	41.21 ± 0.26	< 0.001
Age-year (%)						< 0.001
20–39	35.99 (34.31–37.67)	14.52 (12.84–16.21)	14.73 (13.40–16.06)	26.48 (24.47–28.49)	50.39 (48.74–52.04)	
40–59	37.96 (35.81–40.10)	41.19 (38.99–43.40)	39.57 (38.01–41.14)	38.67 (36.52–40.82)	36.58 (35.22–37.93)	
> = 60	26.05 (24.46–27.64)	44.28 (42.04–46.53)	45.70 (43.98–47.41)	34.85 (32.90–36.80)	13.03 (12.11–13.95)	
Sex (%)						< 0.001
Female	50.90 (48.58–53.22)	43.82 (41.30–46.34)	45.60 (43.95–47.26)	54.27 (52.49–56.04)	52.95 (52.05–53.85)	
Male	49.10 (46.87–51.33)	56.18 (53.66–58.70)	54.40 (52.74–56.05)	45.73 (43.96–47.51)	47.05 (46.15–47.95)	
Race (%)						< 0.001
Non-Hispanic White	66.93 (61.58–72.29)	67.50 (63.66–71.34)	69.98 (66.89–73.06)	67.55 (64.50–70.61)	65.55 (62.82–68.29)	
Non-Hispanic Black	10.53 (9.37–11.69)	15.86 (13.17–18.56)	12.01 (10.12–13.89)	11.49 (9.99–12.99)	8.75 (7.63–9.87)	
Mexican American	8.64 (7.27–10.02)	7.31 (5.52–9.10)	7.02 (5.62–8.42)	7.63 (6.02–9.25)	9.78 (8.18–11.38)	
Others	13.90 (12.78–15.01)	9.33 (7.95–10.71)	11.00 (9.66–12.34)	13.32 (11.74–14.90)	15.92 (14.38–17.45)	
Education (%)						< 0.001
Less than high school	15.63 (14.42–16.83)	17.96 (16.28–19.65)	17.63 (16.04–19.23)	16.09 (14.50–17.68)	14.35 (13.07–15.63)	
High school	22.93 (21.33–24.54)	26.45 (24.25–28.64)	25.56 (24.02–27.10)	24.33 (22.69–25.96)	20.93 (19.67–22.20)	
Above high school	61.44 (58.17–64.71)	55.59 (53.05–58.13)	56.81 (54.73–58.88)	59.58 (57.15–62.01)	64.71 (62.59–66.84)	
Poverty-income ratio (%)						< 0.001
< 1.3	20.12 (18.97–21.28)	23.57 (21.51–25.63)	18.58 (16.94–20.22)	19.58 (17.97–21.18)	20.24 (18.87–21.61)	
1.3–3.5	39.87 (37.82–41.91)	42.75 (40.35–45.16)	40.47 (38.55–42.39)	42.97 (40.91–45.03)	38.14 (36.67–39.61)	
> 3.5	40.01 (37.07–42.95)	33.68 (30.72–36.64)	40.95 (38.53–43.38)	37.45 (34.98–39.93)	41.62 (39.41–43.83)	
Smoking status (%)						< 0.001
Never	55.53 (53.10–57.95)	47.42 (44.84–50.01)	50.73 (48.99–52.47)	52.33 (50.21–54.45)	59.69 (58.17–61.21)	
Now	19.73 (18.49–20.97)	15.65 (13.95–17.36)	16.71 (15.50–17.92)	21.94 (19.99–23.89)	20.81 (19.55–22.08)	
Former	24.74 (23.09–26.39)	36.92 (34.41–39.44)	32.56 (30.77–34.36)	25.73 (24.05–27.41)	19.50 (18.40–20.59)	
Alcohol consumption status (%)						< 0.001
Never	10.99 (10.07–11.91)	11.04 (9.54–12.55)	11.65 (10.42–12.88)	11.20 (10.19–12.21)	10.69 (9.58–11.79)	
Former	12.19 (11.22–13.16)	19.12 (17.19–21.05)	16.07 (14.66–17.48)	14.18 (12.69–15.67)	8.95 (8.20–9.69)	
Mild–Moderate	39.64 (37.67–41.61)	29.55 (27.19–31.90)	33.54 (31.78–35.29)	36.86 (34.97–38.75)	44.48 (42.94–46.03)	
Heavy	37.18 (35.04–39.31)	40.29 (37.96–42.61)	38.74 (36.70–40.79)	37.76 (35.72–39.81)	35.88 (34.43–37.33)	
Marital (%)						< 0.001
Married/Living with Partner	63.72 (60.17–67.28)	63.19 (60.67–65.70)	66.07 (64.17–67.97)	62.38 (60.31–64.45)	63.42 (61.85–64.99)	
Widowed/Divorced/Separated	18.13 (17.16–19.09)	25.24 (23.19–27.28)	23.72 (22.16–25.28)	23.17 (21.54–24.80)	13.26 (12.56–13.96)	
Never married	18.15 (17.05–19.25)	11.58 (10.21–12.95)	10.21 (9.12–11.30)	14.45 (12.85–16.06)	23.32 (21.72–24.92)	
Cancer (%)						< 0.001
No	89.97 (86.05–93.88)	85.24 (83.28–87.20)	85.04 (83.89–86.18)	86.81 (85.47–88.14)	93.57 (93.05–94.08)	
Yes	10.03 (9.34–10.73)	14.76 (12.80–16.72)	14.96 (13.82–16.11)	13.19 (11.86–14.53)	6.43 (5.92–6.95)	
PA (%)						< 0.001
Inactive	21.46 (20.16–22.75)	36.41 (33.74–39.08)	27.80 (26.24–29.37)	22.67 (21.18–24.16)	16.17 (15.19–17.15)	
Low-active	14.27 (13.36–15.17)	16.74 (15.21–18.27)	15.41 (14.18–16.63)	16.33 (14.89–17.78)	12.76 (11.94–13.58)	
Highly active	15.51 (14.67–16.35)	12.95 (11.30–14.60)	15.29 (14.05–16.53)	16.08 (14.69–17.48)	15.85 (15.06–16.65)	
Extremely highly active	48.77 (46.38–51.15)	33.91 (31.72–36.10)	41.50 (39.65–43.35)	44.91 (43.00–46.82)	55.22 (54.09–56.34)	
Waist (cm)	99.34 ± 0.22	126.18 ± 0.28	107.88 ± 0.20	101.43 ± 0.32	90.88 ± 0.17	< 0.001
HDL-C (mmol/L)	1.38 ± 0.01	1.16 ± 0.01	1.29 ± 0.01	1.41 ± 0.01	1.44 ± 0.01	< 0.001
HbA1c (%)	5.64 ± 0.01	6.83 ± 0.04	5.87 ± 0.02	5.64 ± 0.02	5.34 ± 0.01	< 0.001
Uric acid (mg/dL)	5.43 ± 0.01	6.19 ± 0.04	5.89 ± 0.03	5.52 ± 0.03	5.10 ± 0.02	< 0.001
Triglycerides (mmol/L)	153.03 ± 1.38	206.42 ± 4.07	181.46 ± 2.68	159.40 ± 2.46	131.45 ± 1.49	< 0.001
Glucose (mg/dL)	99.67 ± 0.29	136.82 ± 1.44	105.71 ± 0.58	99.59 ± 0.49	90.98 ± 0.19	< 0.001
CKD (%)						< 0.001
No	85.96 (82.00–89.92)	66.83 (64.61–69.06)	77.60 (76.32–78.88)	82.83 (81.44–84.22)	93.31 (92.79–93.83)	
Yes	14.04 (13.28–14.81)	33.17 (30.94–35.39)	22.40 (21.12–23.68)	17.17 (15.78–18.56)	6.69 (6.17–7.21)	
DM (%)						< 0.001
No	87.57 (83.55–91.58)	46.69 (44.57–48.82)	79.03 (77.57–80.49)	89.20 (88.00–90.41)	97.30 (96.96–97.63)	
Yes	12.43 (11.74–13.12)	53.31 (51.18–55.43)	20.97 (19.51–22.43)	10.80 (9.59–12.00)	2.70 (2.37–3.04)	
Hypertension (%)						< 0.001
No	62.42 (59.55–65.29)	2.01 (1.37–2.65)	7.96 (6.84–9.08)	42.56 (40.63–44.49)	98.82 (98.61–99.03)	
Yes	37.58 (35.57–39.59)	97.99 (97.35–98.63)	92.04 (90.92–93.16)	57.44 (55.51–59.37)	1.18 (0.97–1.39)	
CVD (%)						< 0.001
No	91.53 (87.48–95.59)	78.22 (76.31–80.13)	83.84 (82.69–84.99)	89.85 (88.78–90.93)	97.16 (96.78–97.54)	
Yes	8.47 (7.85–9.08)	21.78 (19.87–23.69)	16.16 (15.01–17.31)	10.15 (9.07–11.22)	2.84 (2.46–3.22)	
BMI (%)						< 0.001
<25	29.51 (27.84–31.17)	0.44 (0.23–0.65)	4.32 (3.66–4.98)	30.29 (28.47–32.10)	43.33 (41.86–44.80)	
25–30	33.47 (31.77–35.17)	5.17 (4.13–6.20)	39.18 (37.70–40.66)	28.11 (26.54–29.68)	38.17 (36.95–39.39)	
>30	37.03 (35.04–39.01)	94.40 (93.38–95.41)	56.50 (54.69–58.31)	41.60 (39.78–43.42)	18.50 (17.49–19.51)	
Gout (%)						< 0.001
No	96.05 (91.81–100.28)	88.43 (86.82–90.04)	92.55 (91.70–93.40)	96.01 (95.25–96.77)	98.64 (98.39–98.90)	
Yes	3.95 (3.58–4.33)	11.57 (9.96–13.18)	7.45 (6.60–8.30)	3.99 (3.23–4.75)	1.36 (1.10–1.61)	
Hyperuricemia (%)						< 0.001
No	82.49 (78.82–86.17)	64.14 (61.80–66.48)	71.99 (70.29–73.70)	79.50 (77.97–81.03)	90.43 (89.67–91.19)	
Yes	17.51 (16.46–18.55)	35.86 (33.52–38.20)	28.01 (26.30–29.71)	20.50 (18.97–22.03)	9.57 (8.81–10.33)	

BMI, body Mass Index; DM, diabetes mellitus; PA, physical Activity; CKD, chronic kidney disease; CVD, cardiovascular disease. HbA1c, hemoglobin A1c; HDL-C, high-density lipoprotein cholesterol. Continuous variables are presented as weighted mean ± standard deviation, while categorical variables are presented as weighted percentages (95% confidence interval). Continuous variables were compared by weighted one-way ANOVA, while categorical variables were compared by weighted Rao-Scott chi-square test.

### 3.2 Association between eGDR and HUA

**Figure 2:**
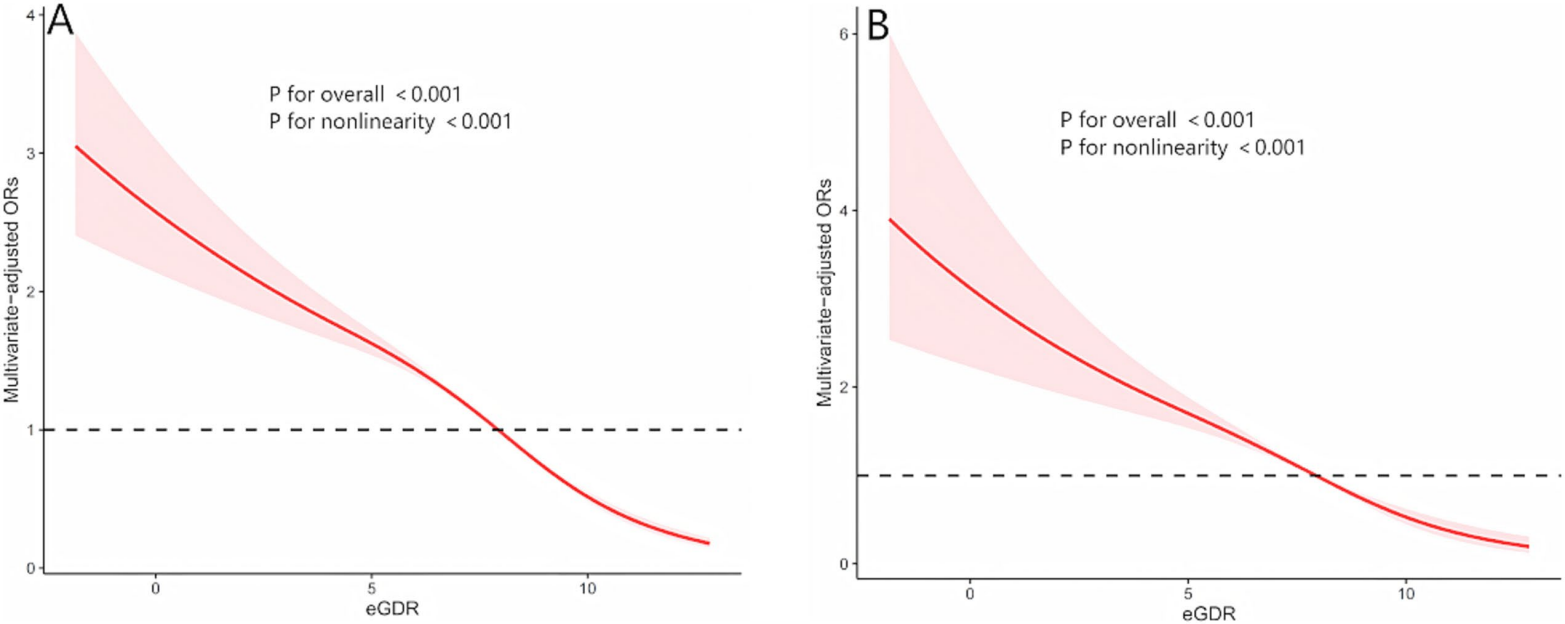
The nonlinear relationships between eGDR with both hyperuricemia and gout by restricted cubic spline fitting. **(A)** eGDR and hyperuricemia; **(B)** eGDR and gout; Adjusted for age, sex, race, body mass index, high-density lipoprotein cholesterol, Triglycerides, Glucose, education, marital, smoking status, alcohol consumption status, poverty-income ratio, physical activity, cancer, diabetes mellitus, cardiovascular disease and chronic kidney disease.

Among 29,340 participants, 5,433 were diagnosed with HUA. Fully adjusted weighted logistic regression models revealed a significant inverse association between eGDR levels and the prevalence of HUA (OR: 0.83, 95% CI: 0.81–0.85, *p* < 0.001), with a 17% reduction in HUA risk for every 1 mg/kg/min increase in eGDR. Further analysis demonstrated that, compared to participants with eGDR < 4 mg/kg/min, the odds ratios (ORs) for HUA in participants with eGDR levels of 4–6 mg/kg/min, 6–8 mg/kg/min, and > 8 mg/kg/min were 0.81 (95% CI: 0.69–0.95; *p* = 0.010), 0.70 (95% CI: 0.58–0.85; *p* < 0.001), and 0.36 (95% CI: 0.29–0.44; *p* < 0.001), respectively (Table 2). Additionally, multivariable-adjusted RCS analysis indicated a significant nonlinear relationship between eGDR and HUA (nonlinear *p*-value < 0.001; Figure 2A).

**Table 2:** Weighted logistic regression coefficients (ORs) and 95% confidence intervals (CIs) for the association between eGDR and hyperuricemia.

eGDR	Case/participants	Model 1		Model 2		Model 3	
		OR (95%CI)	*p* value	OR (95%CI)	*p* value	OR (95%CI)	*p* value
Continuous	5,433/29,340	0.78 (0.77–0.79)	<0.001	0.77 (0.76–0.79)	<0.001	0.83 (0.81–0.85)	<0.001
< 4 (mg/kg/min)	1,178/3,241	Reference		Reference		Reference	
4–6 (mg/kg/min)	1,786/6,257	0.70 (0.60–0.81)	<0.001	0.70 (0.60–0.81)	<0.001	0.81 (0.69–0.95)	0.010
6–8 (mg/kg/min)	1,052/5,347	0.46 (0.40–0.53)	<0.001	0.47 (0.41–0.55)	<0.001	0.70 (0.58–0.85)	<0.001
> 8 (mg/kg/min)	1,417/14,495	0.19 (0.16–0.22)	<0.001	0.19 (0.16–0.22)	<0.001	0.36 (0.29–0.44)	<0.001

OR, Odds Ratio; CI, Confidence interval.

Model 1: unadjusted.

Model 2: Adjusted for age, sex and race.

Model 3: Adjusted for age, sex, race, body mass index, high-density lipoprotein cholesterol, Triglycerides, Glucose, education, marital, smoking status, alcohol consumption status, poverty-income ratio, physical activity, cancer, diabetes mellitus, cardiovascular disease and chronic kidney disease.

### 3.3 Association between eGDR and gout

Among 29,340 participants, 1,346 were diagnosed with gout. As shown in Table 3, a significant inverse association was observed between eGDR levels and the prevalence of gout [odds ratio (OR),95% CI:0.79-0.87, p<0.001], with a 17% reduction in gout risk for every 1 mg/kg/min increase in eGDR. In Model 1 and Model 2, participants with eGDR levels of 4–6 mg/kg/min exhibited a significantly lower risk of gout compared to those with eGDR < 4 mg/kg/min. However, this association was no longer significant in the fully adjusted weig hted logistic regression model (OR: 0.81, 95% CI: 0.63–1.04; *p* = 0.093). In Model 3, compared to participants with eGDR < 4 mg/kg/min, the ORs for gout in participants with eGDR levels of 6–8 mg/kg/min and > 8 mg/kg/min were 0.62 (95% CI: 0.47–0.82; *p* = 0.001) and 0.35 (95% CI: 0.25–0.48; *p* < 0.001), respectively. Additionally, multivariable-adjusted restricted cubic spline (RCS) analysis revealed a significant nonlinear relationship between eGDR and gout (nonlinear *p*-value < 0.001; Figure 2B).

**Table 3:** Weighted logistic regression coefficients (ORs) and 95% confidence intervals (CIs) for the association between eGDR and gout.

eGDR	Case/participants	Model1		Model2		Model3	
		OR (95% CI)	*p* value	OR (95% CI)	*p* value	OR (95% CI)	*p* value
Continuous	1,346/29,340	0.73 (0.71–0.75)	<0.001	0.79 (0.76–0.81)	<0.001	0.83 (0.79–0.87)	<0.001
< 4 (mg/kg/min)	384/3,241	Reference		Reference		Reference	
4–6 (mg/kg/min)	513/6,257	0.62 (0.50–0.76)	<0.001	0.62 (0.50–0.77)	<0.001	0.81 (0.63–1.04)	0.093
6–8 (mg/kg/min)	243/5,347	0.32 (0.24–0.41)	<0.001	0.41 (0.31–0.54)	<0.001	0.62 (0.47–0.82)	0.001
> 8 (mg/kg/min)	206/14,495	0.11 (0.08–0.13)	<0.001	0.20 (0.15–0.25)	<0.001	0.35 (0.25–0.48)	<0.001

OR, Odds Ratio; CI, Confidence interval.

Model 1: unadjusted.

Model 2: Adjusted for age, sex and race.

Model 3: Adjusted for age, sex, race, body mass index, high-density lipoprotein cholesterol, Triglycerides, Glucose, education, marital, smoking status, alcohol consumption status, poverty-income ratio, physical activity, cancer, diabetes mellitus, cardiovascular disease and chronic kidney disease.

### 3.4 Subgroup and sensitivity analyses

Supplementary tables present the results of subgroup analyses, which examined the influence of age, sex, race, and diabetes on the study findings. Detailed results are provided in Supplementary Tables 2, 3. The subgroup analysis for HUA revealed significant interactions between eGDR and age, sex, race, and diabetes. Specifically, in the age subgroup analysis, the association between eGDR and HUA was most pronounced among participants aged 40–59 years (OR: 0.82; 95% CI: 0.79–0.86). In the sex subgroup analysis, the association was more significant in females (OR: 0.78; 95% CI: 0.76–0.81). In the diabetes subgroup analysis, the association was stronger in participants without diabetes (OR: 0.82; 95% CI: 0.80–0.84). For gout, subgroup analysis demonstrated significant interactions between eGDR and age and diabetes. In the age subgroup analysis, the association between eGDR and gout was most significant among participants aged 20–39 years (OR: 0.68; 95% CI: 0.61–0.77). In the diabetes subgroup analysis, the association was more pronounced in participants without diabetes (OR: 0.81; 95% CI: 0.76–0.86). We conducted three sensitivity analyses: the first employed unweighted analysis, the second excluded participants with cancer and CVD, and the third excluded participants with missing covariates. The results of these sensitivity analyses were consistent with the primary findings of the study, as detailed in Supplementary Tables 4–6.

## 4 Discussion

This cross-sectional study is the first to reveal the association between eGDR, a comprehensive indicator of IR, and HUA and gout using nationally representative NHANES data. Our results revealed that, after adjusting for relevant factors, higher eGDR levels were closely linked to a lower prevalence of HUA and gout. Subgroup analyses demonstrated that the relationship between eGDR and HUA differed notably across categories defined by age, sex, race, and diabetes status. In contrast, the association between eGDR and gout showed significant variation only across age and diabetes status groups. Our research findings indicate that eGDR, as a non-invasive comprehensive indicator, can effectively assess IR levels, providing a scientific basis for early intervention in IR, which may help reduce the risk of HUA and gout.

Our findings confirmed the association between eGDR and both HUA and gout, which was consistent with established pathophysiological mechanisms. Previous bidirectional Mendelian randomization studies have demonstrated a causal relationship between IR and both HUA and gout (14). IR influences uric acid metabolism through several mechanisms. Insulin promotes uric acid excretion, but this effect is diminished in the presence of IR (23). Under conditions of high purine load, IR upregulates the expression of urate transporter 1, thereby increasing uric acid reabsorption. Moreover, IR-induced glycolytic dysfunction may contribute to HUA in metabolic syndrome (24). IR induces chronic inflammatory responses in the body, and inflammatory cytokines enhance the expression of xanthine oxidase, leading to increased uric acid production (25). The baseline results of this study indicated that patients with elevated IR levels, as reflected by eGDR, frequently exhibit obesity, hypertension, and dyslipidemia, which could exacerbate uric acid metabolism disorders. In obese patients, adipose tissue released excess free fatty acids and pro-inflammatory factors, contributing to dyslipidemia and a chronic low-grade inflammatory state. Chronic inflammation further promoted the development of IR and upregulates the expression of xanthine oxidase, thus increasing the risk of HUA (26). Studies had demonstrated that adipose tissue IR was significantly associated with an increased risk of HUA (27). HUA was closely associated with skeletal muscle IR, which disrupted insulin and glucose transport into skeletal muscle cells, leading to IR and increasing the risk of HUA in patients (28). Existing studies had thoroughly elucidated the strong association between IR and HUA, providing pathophysiological explanations for the results of this study and further validating the predictive value of eGDR as a comprehensive index for evaluating IR. Notably, eGDR integrated WC, HbA1c, and hypertension indicators, which strongly correlated with the mechanisms underlying HUA. This further explained why eGDR levels are strongly associated with the incidence of HUA and gout in this study.

The results of this study were of considerable value for assessing the risk of HUA and gout. Baseline characteristics of the population revealed that the prevalence of HUA in the eGDR < 4 mg/kg/min group was 35.86%, nearly double the overall prevalence, while the prevalence of gout was 11.57%, nearly three times the overall prevalence. A fully adjusted logistic regression model revealed that, compared to participants with eGDR < 4 mg/kg/min, those with eGDR > 8 mg/kg/min had a significantly reduced probability of developing HUA (OR: 0.36, 95% CI: 0.29–0.44, *p* < 0.001) and gout (OR: 0.35, 95% CI: 0.25–0.48, *p* < 0.001). This suggested that eGDR < 4 mg/kg/min could serve as a high-risk threshold, and using this threshold as a standard for screening high-risk populations for HUA and gout could facilitate early identification and intervention for high-risk individuals. As an integrated indicator for assessing IR levels, combining waist circumference, hypertension, and HbA1c, eGDR offered the advantages of non-invasiveness and reliance on routine clinical parameters, making it particularly suitable for screening populations at high risk for HUA and gout, especially those with comorbidities such as diabetes, hypertension, and obesity. For high-risk individuals with eGDR < 4 mg/kg/min, strengthening the monitoring of uric acid levels was recommended. If abnormal uric acid levels were detected, prompt implementation of appropriate intervention measures was advised. For high-risk individuals with obesity, lifestyle interventions, such as increasing physical activity and improving dietary structure, were employed to reduce weight and thereby improve IR levels. When lifestyle interventions were ineffective, consideration was given to using weight-loss medications or bariatric surgery as alternatives (27). A recent study demonstrated that sodium-glucose cotransporter 2 inhibitors (SGLT2is) significantly reduced serum uric acid levels and lowered the risk of gout by 30–50%. SGLT2is primarily inhibited the flux through the pentose phosphate pathway, thereby reducing purine and uric acid synthesis (29). Furthermore, SGLT2is promoted renal uric acid excretion, thereby lowering serum uric acid concentrations. Moreover, SGLT2is increased urinary glucose excretion, alleviating glucose toxicity and improving insulin sensitivity (30). For patients with type 2 diabetes, SGLT2is significantly reduced IR levels through mechanisms such as weight loss, inhibition of inflammatory responses, and improvement of lipid metabolism, thereby reducing the risk of HUA and gout (31, 32).

Subgroup analysis revealed significant differences in the association between eGDR and HUA across genders, ages, diabetes statuses, and ethnicities. The association between eGDR and gout also showed significant differences across ages and diabetes statuses. Studies indicated that premenopausal women had higher estrogen levels, which promoted renal excretion of uric acid (33). Estrogen also inhibited the activity and expression of xanthine oxidase, thereby reducing uric acid production (34). Hyperinsulinemia caused by IR stimulated androgen synthesis and inhibited estrogen production, thus reducing estrogen’s protective effect against HUA (35). After menopause, the decline in estrogen levels caused uric acid production and excretion to approach those of men (36). However, the decrease in estrogen levels led to significant visceral fat accumulation, resulting in abdominal obesity and increasing the risk of IR. Therefore, whether premenopausal or postmenopausal, the association between eGDR and HUA became more pronounced in women due to the influence of estrogen. IR gradually worsened with age, with the peak incidence of metabolic syndrome occurring between ages 40 and 59, a period when obesity, hypertension, and abnormalities in glucose and lipid metabolism were most prevalent (37, 38). These factors exacerbated uric acid metabolic disorders. In individuals over 60, reduced kidney function may have caused uric acid excretion to be more influenced by the glomerular filtration rate than IR, making the association between eGDR and HUA more pronounced in the 40–59 age group (39, 40). The association between eGDR and HUA/gout was more pronounced in non-diabetic individuals, possibly due to the effects of IR on uric acid synthesis and excretion. In diabetic patients, this mechanism may have been overshadowed by other pathophysiological mechanisms, such as diabetic kidney damage, leading to reduced glomerular filtration rate (GFR) and abnormalities in renal tubular reabsorption and secretion, resulting in elevated uric acid levels (41, 42).

Our study has several significant strengths. First, the sample comprises 29,340 nationally representative adult participants from the United States, which substantially enhances the reliability and generalizability of the findings. Sensitivity analyses were performed to adjust for and evaluate the impact of potential confounders, which further reinforced the robustness of our findings. The study demonstrates a significant nonlinear inverse association between eGDR and the risk of HUA and gout. Additionally, subgroup analyses confirmed the robustness and significance of this association across subgroups defined by gender, age, race, and diabetes status. From a primary care perspective, eGDR, calculated using routine clinical indicators such as hemoglobin A1c, waist circumference, and blood pressure, represents a non-invasive, cost-effective, and efficient alternative for insulin resistance screening. In adult populations, eGDR facilitates the identification of individuals at high risk for HUA and gout. The results of this study indicate that eGDR is a reliable and practical composite marker that provides valuable insights for the prevention and management of HUA and gout.

However, this study still has several potential limitations. First, the cross-sectional study design limits the determination of causality, and future longitudinal studies are needed to validate the findings. Second, although various covariates were adjusted for, the potential influence of certain medications (such as allopurinol) on serum uric acid levels could not be assessed. Third, the eGDR index was measured only at baseline, potentially failing to adequately reflect its dynamic association with HUA and gout incidence. Fourth, the evaluation of certain indicators was based on self-report questionnaires, which may be influenced by recall bias and introduce potential statistical error. Finally, the study sample was limited to U.S. adults, so the results need to be confirmed in other populations with different metabolic risk profiles.

## 5 Conclusion

This study used a nationally representative sample of US adults. It found a significant negative correlation between the IR surrogate marker eGDR and the prevalence of HUA and gout. The results suggest that lower eGDR values are associated with higher rates of HUA and gout. Further analysis using RCS revealed a nonlinear relationship between eGDR and the risk of HUA and gout. Maintaining higher eGDR levels helps reduce the risk of HUA and gout in the general population. This protective effect is particularly pronounced in young people, middle-aged adults, and individuals without diabetes. These results indicate that eGDR serves as a reliable and feasible composite marker with promising clinical utility. Nevertheless, additional research is required to generate robust evidence that elucidates the causal links among eGDR, HUA, and gout.

## Data Availability

The original contributions presented in the study are included in the article/Supplementary material, further inquiries can be directed to the corresponding author.
