# Supplementary material for: Association between higher estimated glucose disposal rate and reduced prevalence of hyperuricemia and gout
Source: Front Nutr. 2025 Sep 23;12:1658286. doi: 10.3389/fnut.2025.1658286 (PMC12501789; doi:10.3389/fnut.2025.1658286)
Supplement: Supplementary file 1 [file Table_1.docx]

Supplementary Material

**Supplementary Table 1** The definitions of hypertension, DM and CKD

**Supplementary Table 2** Subgroup analysis for the association between eGDR and hyperuricemia

**Supplementary Table 3** Subgroup analysis for the association between eGDR and gout

**Supplementary Table 4** The Associations between eGDR and Hyperuricemia/Gout (Unweighted Analysis)

**Supplementary Table 5** The Associations between eGDR and Hyperuricemia/Gout (exclude participants with CVD and cancer)

**Supplementary Table 6** The Associations between eGDR and Hyperuricemia/Gout (exclude participants with missing covariates)

**Supplementary Table 1** The definitions of hypertension, DM and CKD

| **Disease** | **Diagnostic basis** |
| --- | --- |
| DM | A diagnosis by a physician or other health professional |
|  | Random blood sugar ≥11.1 (mmol/L) |
|  | Glycosylated hemoglobin (%) greater than 6.5 |
|  | Use of diabetes medications or insulin |
|  | Fasting plasma glucose (FPG) ≥ 7 mmol/L |
|  | During an oral glucose tolerance test, 2-hour plasma glucose ≥ 11.1 mmol/L |
| Hypertension | A diagnosis by a doctor or other health professional |
|  | An average blood pressure ≥140/90 mmHg |
|  | Use of hypertension medications |
| CKD | Glomerular filtration rate <60 mL/min/1.73 m^2^ |
|  | Urinary albumin to creatinine ratio >30 mg/g |

**Supplementary Table 2** Subgroup analysis for the association between eGDR and hyperuricemia

|  | eGDR | | |
| --- | --- | --- | --- |
|  | OR (95%CI) | p value | P for interaction |
| **Age** |  |  | 0.014 |
| 20-39 | 0.83(0.80,0.87) | <0.001 |  |
| 40-59 | 0.82(0.79,0.86) | <0.001 |  |
| >=60 | 0.84(0.80,0.88) | <0.001 |  |
| **Sex** |  |  | <0.001 |
| Male | 0.87(0.84,0.89) | <0.001 |  |
| Female | 0.78(0.76,0.81) | <0.001 |  |
| **Race** |  |  | 0.046 |
| Non-Hispanic white | 0.83(0.79,0.86) | <0.001 |  |
| Non-Hispanic black | 0.84(0.81,0.87) | <0.001 |  |
| Mexican American | 0.85(0.80,0.91) | <0.001 |  |
| Other | 0.82(0.78,0.86) | <0.001 |  |
| **DM** |  |  | <0.001 |
| Yes | 0.89(0.84,0.94) | <0.001 |  |
| No | 0.82(0.80,0.84) | <0.001 |  |

**Supplementary Table 3** Subgroup analysis for the association between eGDR and gout

|  | eGDR | | |
| --- | --- | --- | --- |
|  | OR (95%CI) | p value | P for interaction |
| **Age** |  |  | 0.007 |
| 20-39 | 0.68(0.61,0.77) | <0.001 |  |
| 40-59 | 0.87(0.80,0.94) | <0.001 |  |
| >=60 | 0.85(0.80,0.90) | <0.001 |  |
| **Sex** |  |  | 0.671 |
| Male | 0.82(0.78,0.87) | <0.001 |  |
| Female | 0.85(0.78,0.93) | <0.001 |  |
| **Race** |  |  | 0.332 |
| Non-Hispanic white | 0.82(0.75,0.90) | <0.001 |  |
| Non-Hispanic black | 0.83(0.78,0.88) | <0.001 |  |
| Mexican American | 0.81(0.70,0.94) | 0.008 |  |
| Other | 0.90(0.84,0.97) | 0.004 |  |
| **DM** |  |  | 0.026 |
| Yes | 0.89(0.83,0.96) | 0.003 |  |
| No | 0.81(0.76,0.86) | <0.001 |  |

|  | Fully adjusted model | | | |
| --- | --- | --- | --- | --- |
|  | hyperuricemia | | gout | |
|  | OR (95%CI) | P value | OR (95%CI) | P value |
| **eGDR** |  |  |  |  |
| Continuous | 0.83(0.81-0.84) | <0.001 | 0.83(0.80-0.86) | <0.001 |
| < 4 (mg/kg/min) | Reference |  | Reference |  |
| 4-6 (mg/kg/min) | 0.80(0.72-0.89) | <0.001 | 0.83(0.70-0.98) | 0.026 |
| 6-8 (mg/kg/min) | 0.65(0.58-0.74) | <0.001 | 0.61(0.50-0.75) | <0.001 |
| > 8 (mg/kg/min) | 0.36(0.32-0.41) | <0.001 | 0.33(0.26-0.41) | <0.001 |

**Supplementary Table 4** The Associations between eGDR and Hyperuricemia/Gout (Unweighted Analysis)

**Supplementary Table 5** The Associations between eGDR and Hyperuricemia/Gout (exclude participants with CVD and cancer)

|  | Fully adjusted model | | | |
| --- | --- | --- | --- | --- |
|  | hyperuricemia | | gout | |
|  | OR (95%CI) | P value | OR (95%CI) | P value |
| **eGDR** |  |  |  |  |
| Continuous | 0.83(0.81,0.85) | <0.001 | 0.80(0.76,0.85) | <0.001 |
| < 4 (mg/kg/min) | Reference |  | Reference |  |
| 4-6 (mg/kg/min) | 0.73(0.60,0.88) | 0.001 | 0.80(0.57,1.12) | 0.186 |
| 6-8 (mg/kg/min) | 0.64(0.52,0.78) | <0.001 | 0.59(0.41,0.85) | 0.005 |
| > 8 (mg/kg/min) | 0.35(0.28,0.43) | <0.001 | 0.29(0.19,0.43) | <0.001 |

**Supplementary Table 6** The Associations between eGDR and Hyperuricemia/Gout (exclude participants with missing covariates)

|  | Fully adjusted model | | | |
| --- | --- | --- | --- | --- |
|  | hyperuricemia | | gout | |
|  | OR (95%CI) | P value | OR (95%CI) | P value |
| **eGDR** |  |  |  |  |
| Continuous | 0.83(0.81,0.85) | <0.001 | 0.82(0.78,0.86) | <0.001 |
| < 4 (mg/kg/min) | Reference |  | Reference |  |
| 4-6 (mg/kg/min) | 0.80(0.67,0.94) | 0.009 | 0.75(0.57,0.98) | 0.036 |
| 6-8 (mg/kg/min) | 0.71(0.58,0.87) | 0.001 | 0.61(0.45,0.81) | 0.001 |
| > 8 (mg/kg/min) | 0.35(0.29,0.43) | <0.0001 | 0.31(0.21,0.45) | <0.0001 |
